# Association of white blood cell count with one-year mortality after cardiac arrest

**DOI:** 10.1016/j.resplu.2024.100816

**Published:** 2024-11-02

**Authors:** Asser M.J. Seppä, Markus B. Skrifvars, Heidi Vuopio, Rahul Raj, Matti Reinikainen, Pirkka T. Pekkarinen

**Affiliations:** a Division of Intensive Care, Department of Anaesthesiology and Intensive Care, University of Helsinki and Helsinki University Hospital, Helsinki, Finland; b Department of Emergency Care and Services, University of Helsinki and Helsinki University Hospital, Helsinki, Finland; c Department of Neurosurgery, University of Helsinki and Helsinki University Hospital, Helsinki, Finland; d Department of Anaesthesiology and Intensive Care, Kuopio University Hospital and University of Eastern Finland, Kuopio, Finland

**Keywords:** Cardiac arrest, Inflammation, Post-cardiac arrest syndrome, Biomarkers, White blood cell

## Abstract

**Introduction:**

Post-resuscitation care of cardiac arrest patients may be complicated by systemic inflammation elicited in response to whole-body ischaemia–reperfusion injury. We assessed the association between early WBC with one-year mortality in a large, unselected population of cardiac arrest patients.

**Methods:**

We collected a retrospective multicentre cohort of ICU-treated CA patients from the Finnish national ICU registry. We used locally estimated scatterplot smoothing (LOESS) curve to assess the association between the most abnormal WBC of the first 24 h in the ICU with the likelihood of death within a year. Multivariable logistic regression analyses were performed to assess the independent association between WBC and one-year mortality. In nested cohort analysis, we tested the association of delay from collapse to return of spontaneous circulation (ROSC) with WBC in linear regression models.

**Results:**

The LOESS curve was U-shaped, with the lowest predicted mortality at 7.5 10^9^/L WBC. Based on this cut-off value, patients were divided into high (≥ 7.5 10^9^/L) and low (< 7.5 10^9^/L) WBC groups. In 4229 patients with high WBC, higher WBC was independently associated with increased one-year mortality (adjusted odds ratio (OR) 1.03 per 10^9^/L, 95 % confidence interval (CI) 1.02–1.04, p < 0.001). In 776 patients with low WBC, lower WBC was independently associated with increased one-year mortality (adjusted OR 0.88 per 10^9^/L, 95 % CI 0.80–0.96, p < 0.001). In a nested cohort analysis, longer ROSC-delay was associated with higher WBC in patients with a shockable rhythm (β = 0.10, R^2^ = 0.04, p < 0.001).

**Conclusions:**

In this large retrospective cohort, WBC was independently associated with one-year mortality after CA. Mortality was lowest in patients with WBC close to the upper limit of the normal reference range. Although WBC is not useful for outcome prognostication in individual patients, our results support the concept of excess inflammation being a harmful component of the post-cardiac arrest syndrome.

## Highlights

• Four out of five cardiac arrest patients have WBC above normal range.
• Mortality after cardiac arrest is lowest in the second quintile of WBC.
• Longer ROSC-delay is associated with elevated white blood cell count.
• Excess inflammation is a harmful component of the post-cardiac arrest syndrome.

## Introduction

Post-resuscitation care of cardiac arrest (CA) patients in the intensive care unit (ICU) is complicated by systemic inflammation resulting from whole-body ischaemia–reperfusion injury (IRI)^1^. Elevated levels of several circulating inflammatory markers, such as interleukin-6 (IL-6) and procalcitonin (PCT) are associated with organ failures and poor long-term patient outcomes^2^, ^3^, ^4^. Leukocytes are fundamental mediators of the inflammatory response and the relative frequencies of leukocyte subclasses, such as neutrophil-to-lymphocyte ratio, have been reported to predict outcome after CA^5^, ^6^, ^7^. A recent study demonstrated the association of higher WBC with increased in-hospital mortality in a single-centre cardiac ICU cohort including CA patients^8^. The rapid onset of whole-body ischaemia, the following ischaemia–reperfusion injury triggering systemic inflammation, and its impact on secondary neurological damage is a unique feature of CA. In the current study, we assess the association of WBC with one-year mortality in CA patients in a multicentre setting. In the current study, we do not aim to build a predictive model for prognostication on the individual patient level but to describe the role of inflammation in post-arrest care.

We collected retrospective registry data from CA patients treated in Finnish university hospital ICUs to assemble a large and unselected cohort of CA patients. Our aim was to: (i) explore the association between WBC recorded during the first 24 h of treatment and one-year mortality in CA patients, (ii) investigate potential differences in the association of WBC with mortality across varying levels of WBC, and (iii) test whether WBC correlates with the time from collapse to return of spontaneous circulation (ROSC) in patients with shockable or non-shockable initial rhythm. Our hypothesis was that elevated WBC levels would reflect the magnitude of inflammation in response to IRI and associate with one-year mortality. Furthermore, we hypothesised that the association between WBC and mortality would not be linear. We also hypothesised that longer ROSC-delay would lead to more severe IRI reflected as a correlation between ROSC-delay and WBC.

## Materials and Methods

### Study design

This was a retrospective analysis of a multicentre cohort consisting of unselected CA patients (including both in- and out-of-hospital CA) treated in five university hospitals in Finland. These centres serve a population of 3.2 million, covering 60 % of the entire population of Finland. We collected data on demographics, in-hospital measurements, self-care independence pre-arrest, and one-year mortality. We also performed a nested cohort analysis on data from one of the centres (Helsinki University Hospital), where additional data on initial rhythm and ROSC-delay were available.

The ethical approval for this study was granted by the Finnish Institute for Health and Welfare (THL/1298/5.05.00/2019, “Long-term mortality of patients with acute brain injuries treated in Finnish university hospital intensive care units”) on November 7th 2019 and the Operative Division of Helsinki University Hospital (194/13/03/02/2014 TMK02§97, “Long-term mortality of patients with acute brain injuries treated in Finnish university hospital intensive care units”) in June 2014. The study was conducted in accordance with the ethical standards of the responsible institutes and the 1975 Helsinki Declaration.

### Database and data collection

We extracted patient data from the Finnish Intensive Care Consortium (FICC) database^9^. The FICC database is used for ICU benchmarking in Finland. In-hospital data are collected to the database automatically via monitors and laboratory systems and subsequently validated locally by trained personnel. Diagnoses and pre-hospital data are entered manually. The FICC database is a general ICU registry and, therefore, does not include CA-specific variables, such as initial rhythm or ROSC delay. We included all adult (≥ 18 years) CA patients recorded in the FICC database with admission dates between January 1st 2003 and December 31st 2019. CA treatment practices in Finnish ICUs have largely been similar at a certain time, but naturally practice has evolved during the inclusion time frame. In this study, we defined CA as an ICU admission diagnosis of “post cardiac arrest” recorded in Acute Physiology and Chronic Health Evaluation II (APACHE II)^10^. We retrieved information on vital status at one year after admission from the Finnish Population Information System and all other variables from the FICC. For inclusion, we required complete data for WBC, age, sex, APACHE II score^10^, highest Sequential Organ Failure Assessment (SOFA) score recorded during the first 24 h of treatment^11^, all SOFA sub-scores (except liver sub-score, which was assumed normal if missing), admission date and day of death or vital status at one-year after admission. Additionally, we retrieved from FICC a simplified World Health Organization-Eastern Cooperative Oncology Group (WHO/ECOG) classification of the premorbid physical status, where “independent” is defined as the patient being independent in self-care and “dependent” is defined as the patient being partly or fully dependent on help in self-care prior to hospitalisation^12^. We used WBC recorded as the most abnormal value during the first 24 h of treatment, originally collected for calculating APACHE II score in the FICC database. The normal reference values in Finland for the clinical laboratory WBC measurement used are 3.4–8.2 10^9^/L. The complete list of variables extracted from the FICC database is presented in Table 1.

**Table 1:** Baseline characteristics of the study population.

**Patient characteristics**	**One-year survivors** (n = 2256)	**One-year non-survivors** (n = 2749)
Age, years	63 (54–71)	67 (58–75)
Male sex, n (%)	1750 (75.6 %)	2029 (73.8 %)
WBC, 10^9^/L	11.4 (8.6–15.0)	12.5 (9.0–16.6)
Independent in self-care pre-arrest, n (%)	1991 (88.3 %)	2085 (75.8 %)
APACHE II score	25 (19–30)	32 (27–38)
APACHE II score without WBC points	25 (19–30)	32 (27–37)
SOFA score	9 (7–11)	11 (9–13)
Respiration SOFA sub-score	2 (2–3)	3 (2–3)
CNS SOFA sub-score	3 (0–4)	4 (3–4)
Renal SOFA sub-score	0 (0–0)	0 (0–2)
Cardiovascular SOFA sub-score	3 (3–4)	4 (3–4)
Coagulation SOFA sub-score	0 (0–1)*	0 (0–1)*
Liver SOFA sub-score	0 (0–0)*	0 (0–0)*

Data presented as medians (inter-quartile ranges) or as absolute numbers (percentages). Abbreviations: APACHE: Acute Physiology and Chronic Health Evaluation; CA: cardiac arrest; CNS: central nervous system; SOFA: Sequential Organ Failure Assessment; WBC: white blood cell count.

*values were higher in one-year non-survivors.

We performed additional analyses on a nested cohort consisting of patients admitted between January 1st 2003 and December 31st 2013 in one (Helsinki University Hospital) of the five centres included in the full data. In the nested cohort, data on delay from collapse to ROSC and initial rhythm (shockable/non-shockable) were also available.

### Statistical analysis

We presented continuous data as medians and interquartile ranges. None of the variables followed a normal distribution and thus we used Mann Whitney *U* test to assess statistical significance. We used chi-square test for categorical variables.

We aimed to select the smallest set of available variables that would give sufficient adjustment for mortality risk related to acute physiological derangements, comorbidities, and patient characteristics to be used in multivariable regression models. We chose the 24-hour SOFA score as the measure of acute physiological derangement since it is widely used measure for multiple organ failure. We have previously shown that 24-hour SOFA score (also when the neurological component is excluded) is strongly associated with one-year outcome after CA^13^. We did not include APACHE II score since it overlaps with SOFA. We chose independency in self-care (simplified WHO/ECOG classification) as a robust measure of severity of comorbidities and age and sex as basic patient characteristics.

To give a visual overview of the population, we divided it into quintiles based on WBC, and calculated mortality for each quintile. We used chi-square test to assess differences in mortality between the quintiles.

We used a multivariable logistic regression model including age, sex, 24-hour SOFA score and premorbid independence in self-care to determine predicted mortality, which we then plotted against corresponding WBC values. We then fitted a locally estimated scatterplot smoothing (LOESS) curve to assess the relationship between WBC and predicted mortality at different values of WBC. Again, we used multivariable logistic regression analyses adjusted for the same covariates (age, sex, 24-hour SOFA score and premorbid independence in self-care) to assess the independent association of WBC and outcome separately in high and low WBC groups. Also, we conducted a sensitivity analysis in the same manner in three different WBC groups: WBC < 3.4 10^9^/L, WBC 3.4–8.2 10^9^/L (normal range of WBC in participating laboratories) and WBC > 8.2 10^9^/L. We conducted a nested cohort analysis on patients with data on initial rhythm and ROSC-delay available. In the nested cohort analyses, we used univariate linear regression analysis to assess the relationship between ROSC-delay and WBC separately in patients with shockable rhythm (ventricular fibrillation or ventricular tachycardia) and in patients with non-shockable rhythm (asystole or pulseless electrical activity). All statistical analyses were performed using R (version 4.0.3, R Foundation for Statistical Computing, Vienna, Austria).

## Results

**Fig. 1:**
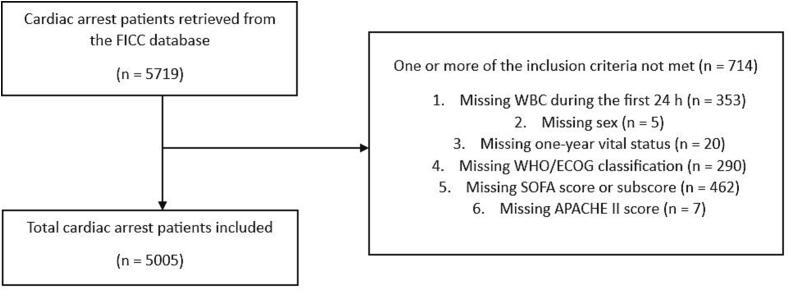
Patient inclusion flow chart. Abbreviations: APACHE: Acute Physiology and Chronic Health Evaluation; FICC: Finnish Intensive Care Consortium; SOFA: Sequential Organ Failure Assessment; WBC: white blood cell count; WHO/ECOG: World Health Organization-Eastern Cooperative Oncology Group.

**Fig. 2:**
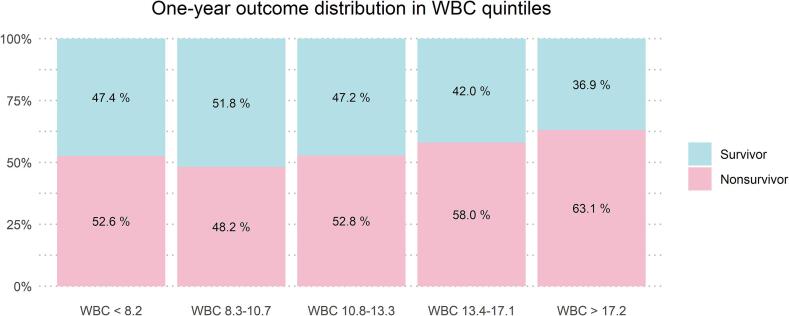
One-year mortality in WBC quintiles. The unit for WBC values is 10^9^/L. Differences in mortality between quintiles were significant (p < 0.001). WBC, white blood cell count.

In total, 5005 patients were included (Fig. 1). One-year mortality was 54.9 %. Non-survivors were older and had higher WBCs, 24-hour SOFA scores, SOFA sub-scores, and APACHE II scores than survivors. The baseline characteristics of both groups are presented in Table 1. Differences in mortality between WBC quintiles were statistically significant (p < 0.001). One-year mortality was highest (63.1 %) in the fifth WBC quintile (WBC > 17.2 10^9^/L) and lowest (48.2 %) in the second quintile (WBC 8.3–10.7 10^9^/L) (Fig. 2).

### Association between WBC and predicted mortality

**Fig. 3:**
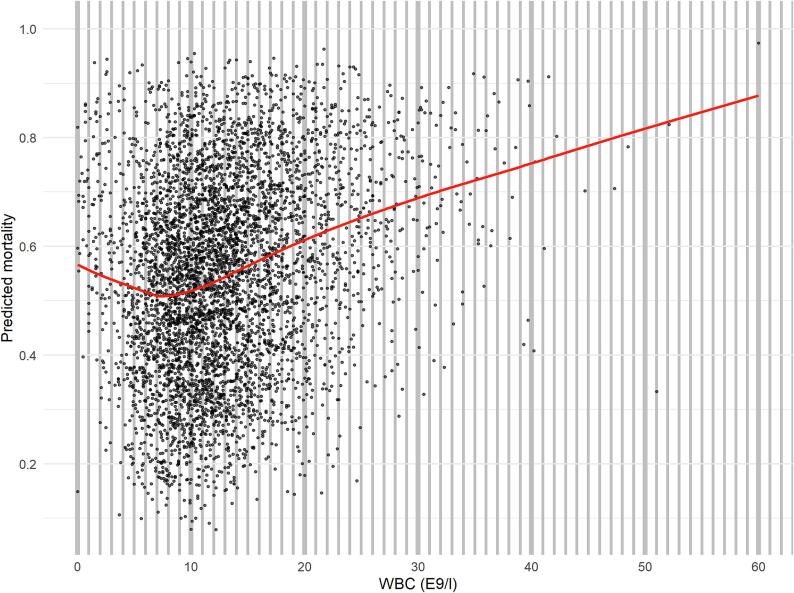
LOESS curve of the association between WBC and predicted mortality. The lowest likelihood of death was recorded at WBC value of 7.5 10^9^/L. Multivariable logistic regression analysis including age, sex, SOFA score, independence in self-care and WBC was used to determine predicted mortality. Outliers extending beyond WBC 60 10^9^/L (n = 2) are not shown. Abbreviations: LOESS: locally estimated scatterplot smoothing; SOFA: Sequential Organ Failure Assessment; WBC: white blood cell count.

The lowest point of the LOESS curve was at 7.5 WBC 10^9^/L, meaning lowest predicted mortality at this level of WBC (Fig. 3). The curve was U-shaped with a consistent increase in mortality with both lower and higher WBC values (Fig. 3). We divided the cohort into high (WBC ≥ 7.5 10^9^/L), and low (WBC < 7.5 10^9^/L) WBC groups based on this cut-off value.

### Association between WBC and mortality in high and low WBC groups

4229 patients had WBC ≥ 7.5 10^9^/L and 776 patients had WBC < 7.5 10^9^/L. We used multivariable logistic regression including age, sex, WBC, premorbid independence in self-care and SOFA score to determine the association of these factors with one-year mortality separately in the high WBC group (Table 2) and the low WBC group (Table 3). In both groups, advanced age, higher 24-hour SOFA score and not being independent in self-care were associated with higher one-year mortality. In the high WBC group, higher WBC was associated with increased mortality (adjusted odds ratio (OR) 1.03 per 10^9^/L, 95 % confidence interval (CI) 1.02–1.04, p < 0.001). Conversely, in the low WBC group, lower WBC was associated with increased mortality (adjusted OR 0.88 per 10^9^/L, 95 % CI 0.80–0.96, p < 0.001).

**Table 2:** Univariate and multivariable logistic regression models for one-year mortality in cardiac arrest patients in the high WBC group (WBC ≥ 7.5 10^9^/L, n = 4229).

**Patient characteristics**	**Univariate logistic regression**		**Multivariable logistic regression**	
	**Odds ratio (95 % CI)**	**p-value**	**Odds ratio (95 % CI)**	**p-value**
Age (years)	1.02 (1.02–1.03)	< 0.001	1.02 (1.01–1.02)	< 0.001
Male sex	0.87 (0.75–1.00)	0.043	0.84 (0.72–0.98)	0.026
WBC (10^9^/L)	1.04 (1.03–1.05)	< 0.001	1.03 (1.02–1.04)	< 0.001
Not independent in self-care	2.34 (1.98–2.77)	< 0.001	1.82 (1.53–2.18)	< 0.001
SOFA score (points)	1.24 (1.21–1.26)	< 0.001	1.21 (1.19–1.24)	< 0.001

Abbreviations: CI: confidence interval; SOFA: Sequential Organ Failure Assessment; WBC: white blood cell count.

**Table 3:** Univariate and multivariable logistic regression models for one-year mortality in cardiac arrest patients in the low WBC group (WBC < 7.5 10^9^/L, n = 776).

**Patient characteristics**	**Univariate logistic regression**		**Multivariable logistic regression**	
	**Odds ratio (95 % CI)**	**p-value**	**Odds ratio (95 % CI)**	**p-value**
Age (years)	1.01 (1.00–1.03)	0.008	1.02 (1.01–1.03)	0.002
Male sex	1.21 (0.87–1.68)	0.254	1.11 (0.77–1.61)	0.573
WBC (10^9^/L)	0.80 (0.74–0.87)	< 0.001	0.88 (0.80–0.96)	0.007
Not independent in self-care	2.76 (1.81–4.30)	< 0.001	2.66 (1.66–4.38)	< 0.001
SOFA score (points)	1.30 (1.24–1.37)	< 0.001	1.29 (1.22–1.36)	< 0.001

Abbreviations: CI: confidence interval; SOFA: Sequential Organ Failure Assessment; WBC: white blood cell count.

### Sensitivity analysis

As a sensitivity analysis, we repeated the multivariable logistic regression analyses with three groups of WBC: below the normal range (< 3.4 10^9^/L, n = 127), within the normal range (3.4–8.2 10^9^/L, n = 916) and above the normal range (> 8.2 10^9^/L, n = 3 962) of the WBC laboratory test. Notably, almost four out of five patients had WBC above the normal range and higher WBC was associated with increased mortality in this group (adjusted OR 1.03 per 10^9^/L, 95 % CI 1.02–1.04, p < 0.001). In the groups with WBC < 3.4 10^9^/L and WBC 3.4–8.2 10^9^/L the adjusted ORs for WBC were below one but did not reach statistical significance (Supplemental material, Tables 1–3).

### Nested cohort

Data on initial rhythm (shockable/non-shockable) were available for 1085 patients in the nested cohort of patients treated in one of the five centres (Helsinki University Hospital). Of these, 692 patients had a shockable initial rhythm, and 393 patients had a non-shockable rhythm. Patients with a non-shockable rhythm had higher one-year mortality (62.1 % vs 33.8 %, p < 0.001) and a shorter ROSC-delay (11.0 min vs 18.0 min, p < 0.001) than patients with a shockable rhythm. WBC levels were comparable between non-shockable and shockable groups (11.4 10^9^/L vs 11.8 10^9^/L, p = 0.283).

In patients with a shockable rhythm, ROSC-delay was associated with WBC (β = 0.10, R^2^ = 0.04, p < 0.001) in a univariate linear regression model. Conversely, in patients with a non-shockable rhythm ROSC-delay was not associated with WBC (β = 0.02, R^2^ = 0.00, p = 0.528) (Supplemental material, Fig. 1). In patients with a shockable rhythm, the association of ROSC-delay with WBC remained statistically significant in a multivariable linear regression model adjusting for age, sex, premorbid independence in self-care and SOFA score (β = 0.09, p < 0.001) (Supplemental material, Table 4).

In a multivariable logistic regression analysis including initial shockable rhythm and ROSC-delay, higher WBC was not associated with increased mortality (adjusted OR 0.99 per 10^9^/L, 95 % CI 0.97–1.02, p < 0.492) (Supplemental material, Table 5).

## Discussion

This was a retrospective multicentre analysis of the association of WBC with one-year mortality in adult CA patients treated in the ICU, using the most abnormal WBC value during the first 24 h of care. Mortality was significantly different between WBC quintiles, being lowest in the second quintile and highest in the fifth quintile. In a model adjusting for relevant baseline variables and the SOFA score, we discovered an independent, U-shaped association between WBC and one-year mortality. We identified WBC 7.5 10^9^/L as a turning point in this cohort, above which higher WBC was independently associated with increased mortality. This observation was confirmed in the sensitivity analysis using the upper limit of WBC normal range (8.2 10^9^/L) as cut-off point. On the contrary, in patients with WBC < 7.5 10^9^/L, lower WBC was independently associated with increased mortality, however this association was less clear since it did not reach statistical significance in the sensitivity analysis in groups with WBC within or below normal range. Our results are in line with previous findings of higher WBC being detrimental in cardiac ICU patients^8^. Our results build on these data focusing on CA patients in a multicentre setting. While there is considerable overlap in the outcome distribution over different values of WBC and individualized clinical decision making cannot be based on WBC, our findings emphasise adverse outcomes in patients with profound inflammation after CA. Systemic inflammation after cardiac arrest is of specific interest since it has an impact on the secondary neurological damage occurring after ROSC and provides a potential treatment target^14^. Furthermore, we found that longer ROSC-delay was associated with higher WBC in patients with a shockable rhythm in nested cohort analysis.

Our findings support the presence of an inflammatory component in post-cardiac arrest syndrome^15^. Interestingly, lowest mortality was associated with WBC close to the upper limit of normal reference values. It is possible that moderate leukocytosis after CA reflects a physiological reaction to the extreme stress caused by whole-body ischaemia. Although excessive inflammation is harmful after CA^6^, ^7^, ^2^, ^3^, ^4^, also failure of the body to recruit leukocytes into circulation may be a sign of impaired physiological resources, for example because of an underlying acute or chronic comorbidity leading to an immunocompromised state. In this regard, WBC differs from other inflammatory markers such as C-reactive protein and procalcitonin, which have very low or undetectable normal values and any elevation is interpreted as abnormal^16^, ^17^. It is possible, however, that systemic inflammation after CA is harmful only above a certain threshold. In patients with sepsis, an early hypoinflammatory state leads to decreased survival^18^. Comparable findings can be expected in CA patients, considering the similarities between inflammatory responses of sepsis and CA^1^. A long-term goal is to identify a possible immunomodulatory therapy that would ultimately improve patient outcomes. Early high-dose methylprednisolone has been shown to reduce haemodynamic instability and markedly decrease IL-6 levels in out-of-hospital CA (OHCA) patients^14^, ^19^. Methylprednisolone also caused leukocytosis and did not significantly decrease long-term mortality or neurological outcome scores^14^. Also, tocilizumab administration has been associated with reduced haemodynamic instability and reduced cardiac damage after CA^20^, ^21^. To date, there is no evidence that anti-inflammatory treatment reliably reduces mortality or improves neurological outcome.

Currently, neuron-specific enolase is the only biomarker in clinical use for post-resuscitation outcome prognostication^22^, ^23^. Inflammatory markers have some predictive value on the population level^2^ but their use in prognostication on the single patient level is hindered by large overlap between outcome groups. This is true also for WBC, but as a readily available marker, WBC could possibly be used in multifactorial scoring systems and machine learning models to aid in clinical decision-making. For example, the SLANT score comprising WBC, initial rhythm, bystander resuscitation, epinephrine dose and ROSC-delay has been reported to predict outcome after OHCA^24^.

In our study, the association between WBC and ROSC-delay was observed only in patients with a shockable initial rhythm. Since cardiac etiology is the most common cause of CA in this group, it is likely that whole-body ischaemia began abruptly in these patients^25^. Conversely, patients with non-shockable rhythm likely comprise a more heterogenous group in which there is a greater proportion of patients with precipitating inflammatory pathology (e.g., septic shock, pneumonia, COPD exacerbation) already pre-arrest. Thus, it is possible that the influence of CA on inflammation differs between these two groups. The association between ROSC-delay and WBC in patients with a shockable rhythm is plausible explanation for the lack of independent association of WBC with mortality in a multivariable logistic regression analysis including ROSC-delay and initial rhythm. It appears that, at least to some extent, the magnitude of inflammation is associated with the extent of ischaemic damage, but its significance in the pathologic process after CA is unclear.

## Limitations

Although our findings are based on a large multicentre registry with long outcome follow-up time, several limitations should be considered. Our study was limited by retrospective data with missing information on other inflammatory markers such as CRP (C-reactive protein). Confounding etiologies for WBC elevations such as concomitant infections are also not recorded in the registry. Additionally, important parameters regarding CA event such as initial rhythm and ROSC-delay were not available for most of the patients. The data consists of an unselected population of CA patients including both out-of-hospital and in-hospital CA patients. Also, our data on WBC was limited to a single most abnormal value measured during the first 24 h of care. In clinical setting, WBC trajectories over several days might be more useful in assessing the magnitude of inflammatory reaction after CA.

## Conclusions

In this large multicentre study of unselected ICU-treated CA patients, the WBC level measured during the first 24 h of ICU treatment was independently associated with one-year mortality. Mortality was lowest in patients with WBC close to the upper limit of the normal reference range. Although WBC is not useful for outcome prognostication in individual patients, our results support the concept of excess inflammation being a harmful component of the post-cardiac arrest syndrome.

## CRediT authorship contribution statement

**Asser M.J. Seppä:** Writing – review & editing, Writing – original draft, Visualization, Investigation, Formal analysis. **Markus B. Skrifvars:** Writing – review & editing, Supervision, Funding acquisition, Conceptualization. **Heidi Vuopio:** Writing – review & editing, Formal analysis. **Rahul Raj:** Writing – review & editing, Data curation. **Matti Reinikainen:** Writing – review & editing, Data curation. **Pirkka T. Pekkarinen:** Writing – review & editing, Supervision, Methodology, Funding acquisition, Conceptualization.

## Funding

This study was funded by Finska Läkaresällskapet, Medicinska Understödsföreningen Liv och Hälsa, Einar och Karin Stroems stiftelse and Kirsti och Tor Johanssons Hjärt- och Cancerstiftelse.

## Declaration of competing interest

The authors declare that they have no known competing financial interests or personal relationships that could have appeared to influence the work reported in this paper.
