# Supplementary material for: Association of white blood cell count with one-year mortality after cardiac arrest
Source: Resusc Plus. 2024 Nov 2;20:100816. doi: 10.1016/j.resplu.2024.100816 (PMC11565414; doi:10.1016/j.resplu.2024.100816)
Supplement: Supplementary Data 1 [file mmc1.docx]

Supplemental table 1. Univariate and multivariable logistic regression models for one-year mortality in cardiac arrest patients with WBC < 3.4 10^9^/L (n = 127).

| **Patient characteristics** | **Univariate logistic regression** | | **Multivariable logistic regression** | |
| --- | --- | --- | --- | --- |
|  | **Odds ratio (95% CI)** | **p-value** | **Odds ratio (95% CI)** | **p-value** |
| Age (years) | 1.03 (1.00–1.06) | 0.048 | 1.04 (1.02–1.08) | 0.017 |
| Male sex | 1.18 (0.49–2.72) | 0.704 | 0.64 (0.22–1.75) | 0.404 |
| WBC (10^9^/L) | 0.66 (0.40–1.04) | 0.080 | 0.71 (0.40–1.24) | 0.237 |
| Not independent in self-care | 3.42 (0.91–22.4) | 0.113 | 2.92 (0.61–22.2) | 0.223 |
| SOFA score (points) | 1.29 (1.14–1.49) | < 0.001 | 1.34 (1.16–1.58) | < 0.001 |

Abbreviations: CI: confidence interval; SOFA: Sequential Organ Failure Assessment; WBC: white blood cell count

Supplemental table 2. Univariate and multivariable logistic regression models for one-year mortality in cardiac arrest

patients with WBC 3.4–8.2 10^9^/L (n = 916).

| **Patient characteristics** | **Univariate logistic regression** | | **Multivariable logistic regression** | |
| --- | --- | --- | --- | --- |
|  | **Odds ratio (95% CI)** | **p-value** | **Odds ratio (95% CI)** | **p-value** |
| Age (years) | 1.02 (1.01–1.03) | < 0.001 | 1.02 (1.01–1.03) | 0.004 |
| Male sex | 1.16 (0.85–1.58) | 0.356 | 1.04 (0.74–1.47) | 0.811 |
| WBC (10^9^/L) | 0.93 (0.84–1.04) | 0.195 | 0.92 (0.83–1.04) | 0.191 |
| Not independent in self-care | 3.06 (2.11–4.50) | < 0.001 | 2.94 (1.96–4.49) | < 0.001 |
| SOFA score (points) | 1.28 (1.22–1.34) | < 0.001 | 1.28 (1.22–1.35) | < 0.001 |

Abbreviations: CI: confidence interval; SOFA: Sequential Organ Failure Assessment; WBC: white blood cell count

Supplemental table 3. Univariate and multivariable logistic regression models for one-year mortality in cardiac arrest patients with WBC > 8.2 10^9^/L (n = 3962).

| **Patient characteristics** | **Univariate logistic regression** | | **Multivariable logistic regression** | |
| --- | --- | --- | --- | --- |
|  | **Odds ratio (95% CI)** | **p-value** | **Odds ratio (95% CI)** | **p-value** |
| Age (years) | 1.02 (1.02–1.03) | < 0.001 | 1.02 (1.01–1.02) | < 0.001 |
| Male sex | 0.87 (0.75–1.01) | 0.061 | 0.85 (0.73–0.99) | 0.036 |
| WBC (10^9^/L) | 1.04 (1.03–1.06) | < 0.001 | 1.03 (1.02–1.04) | < 0.001 |
| Not independent in self-care | 2.27 (1.91–2.70) | < 0.001 | 1.75 (1.46–2.10) | < 0.001 |
| SOFA score (points) | 1.23 (1.21–1.26) | < 0.001 | 1.21 (1.18–1.24) | < 0.001 |

Abbreviations: CI: confidence interval; SOFA: Sequential Organ Failure Assessment; WBC: white blood cell count

Supplemental table 4. Multivariable linear regression model for predictors of WBC in patients with a shockable rhythm (n = 672).

|  | **B (unstandardized beta)** | **p-value** |
| --- | --- | --- |
| ROSC (min) | 0.09 | < 0.001 |
| SOFA score (points) | 0.21 | 0.007 |
| Not independent in self-care | 1.02 | 0.268 |
| Female sex | 1.27 | 0.012 |
| Age (years) | -0.04 | 0.010 |

Abbreviations: ROSC: return of spontaneous circulation; SOFA: Sequential Organ Failure Assessment

Supplemental table 5. Univariate and multivariable logistic regression models for one-year mortality in cardiac arrest patients with available data on ROSC-delay and initial rhythm (n = 1035).

| **Patient characteristics** | **Univariate logistic regression** | | **Multivariable logistic regression** | |
| --- | --- | --- | --- | --- |
|  | **Odds ratio (95% CI)** | **p-value** | **Odds ratio (95% CI)** | **p-value** |
| Age (years) | 1.03 (1.02–1.03) | < 0.001 | 1.03 (1.02–1.04) | < 0.001 |
| Male sex | 0.96 (0.72–1.27) | 0.761 | 0.99 (0.72–1.36) | 0.936 |
| WBC (10^9^/L) | 1.01 (0.99–1.03) | 0.440 | 0.99 (0.97–1.02) | 0.492 |
| Not independent in self-care | 3.49 (2.16–5.81) | < 0.001 | 2.49 (1.46–4.34) | < 0.001 |
| SOFA score (points) | 1.27 (1.22–1.33) | < 0.001 | 1.21 (1.15–1.27) | < 0.001 |
| Shockable rhythm | 0.32 (0.24–0.41) | < 0.001 | 0.35 (0.26–0.49) | < 0.001 |
| ROSC-delay (min) | 1.02 (1.00–1.03) | 0.007 | 1.05 (1.03–1.06) | < 0.001 |

Abbreviations: CI: confidence interval; SOFA: Sequential Organ Failure Assessment; ROSC: return of spontaneous circulation; WBC: white blood cell count


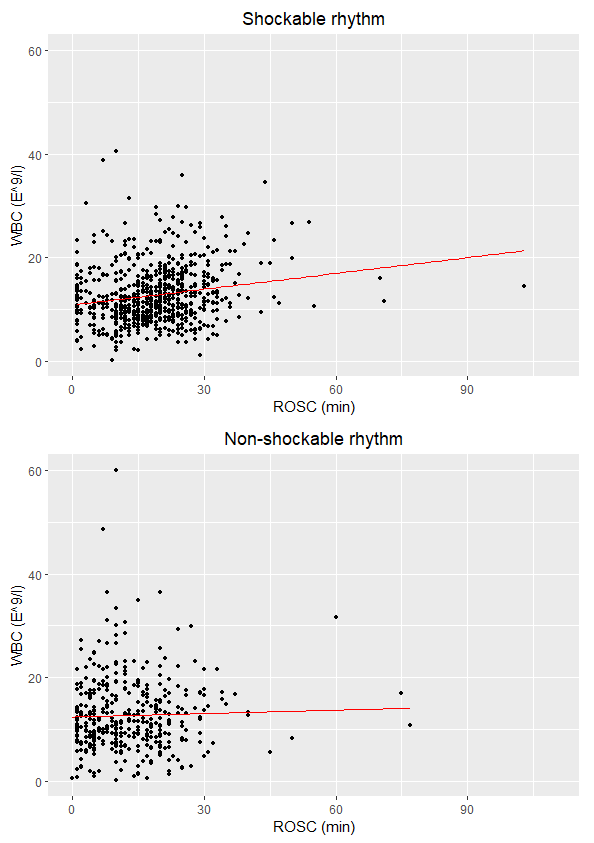


Supplemental figure 1. Univariate linear regression model between ROSC-delay and WBC in patients with shockable (β = 0.10, R2 = 0.04, p < 0.001) and non-shockable rhythms (β = 0.02, R2 = 0.00, p = 0.528). Abbreviations: ROSC: return of spontaneous circulation; WBC: white blood cell count
